# Validation of reoperations due to infection in the Swedish Hip Arthroplasty Register

**DOI:** 10.1186/1471-2474-15-384

**Published:** 2014-11-19

**Authors:** J Viktor Lindgren, Max Gordon, Per Wretenberg, Johan Kärrholm, Göran Garellick

**Affiliations:** Department of Molecular Medicine and Surgery, Section of Orthopaedics, Karolinska Institutet, Stockholm, Sweden; Swedish Hip Arthroplasty Register, Registercentrum VGR, Gothenburg, Sweden; Department of Orthopaedics, Institute of Clinical Sciences at Sahlgrenska Academy, University of Gothenburg, Gothenburg, Sweden; Department of Clinical Sciences, Danderyd Hospital, Karolinska Institutet, Stockholm, Sweden; Department of Orthopaedics, Karolinska University Hospital, Stockholm, Solna SE-171 76 Sweden

**Keywords:** Arthroplasty Register, Validity, Completeness, Hip replacement, Reoperation, Infection

## Abstract

**Background:**

Complete or almost complete recording of reoperations is essential to enable a correct interpretation of data in arthroplasty registers. The completeness of recordings due to infection is unknown in the Swedish Hip Arthroplasty Register (SHAR). We therefore used a combination of data from the Swedish Prescribed Drug Register (SPDR) and studies of medical records to validate the data of reoperations due to infection in the SHAR.

**Methods:**

All patients registered for a primary Total Hip Replacement (THR) in the SHAR between July 1, 2005 and December 31, 2008 were selected for the study (45,531 patients with 49,219 THRs) and were matched with the SPDR. All patients with a minimum of 4 weeks of continuous outpatient antibiotic treatment within 2 years after their primary THR (1,989 patients, with 2,219 THRs) were selected for a medical records review to find the THRs reoperated due to infection.

**Results:**

599 (1.3%) of the THRs had been reoperated within 2 years after the index operation and in 47.4% of these the prosthesis had been revised or extracted. 400 of the THRs were registered for a reoperation in the SHAR resulting in a completeness of 67%.

**Conclusions:**

The completeness of registration due to early infection after THR questions whether the SHAR reoperation data can be used in order to evaluate changes in postoperative infection rates.

**Electronic supplementary material:**

The online version of this article (doi:10.1186/1471-2474-15-384) contains supplementary material, which is available to authorized users.

## Background

National arthroplasty register data is often used to evaluate the outcome after hip replacement surgery. The data can be used in order to evaluate risks of revision surgery, the patient reported outcome, mortality and more. The strengths of register data are the large number of patients available for inclusion making statistical analyses robust when evaluating rare competing events, but it assumes high validity of the data. Validation of national arthropasty register data is often done by matching data from health care registries, by investigating medical journals or by questionnaires answered by patients [1]. Previous studies have demonstrated that the overall validity of revision surgery is high in many arthroplasty registries [2–4].

Arthroplasty register data has been used to estimate infection risks after replacement surgery and it has been postulated that the risk of infection is increasing [5–7]. However, concerns have been raised by Jämsen et al. regarding the validity of recordings of revision due to infection in the Finnish Arthroplasty Register, and there is reason to believe that the same might apply to other registries as well [8].

As part of the ambition to continuously increase the quality of data in the Swedish Hip Arthroplasty Register (SHAR), we aimed to evaluate the validity of reoperations due to infection. By matching the data in SHAR with the Swedish Prescribed Drug Register (SPDR) we conducted a targeted study of medical records to find non recorded reoperations due to infection.

## Methods

The SHAR was started in 1979 and all private and public orthopaedic units in Sweden voluntary participate. 98% of all primary Total Hip Replacements (THRs) in Sweden are reported to the register [9]. The register holds information including personal identification number, age, sex, side, diagnosis and a number of operative details regarding surgical approach, implant, fixation method etc. Any type of reoperation as well as the reason for the surgery is to be reported to the register. The SHAR is connected to the Swedish Death Register, thereby also including survival time of implant and patient.

In the SHAR reoperation denotes any kind of subsequent surgery of adjacent tissues related to the primary THR, whereas revision denotes a reoperation including exchange of parts or entire prosthesis, or extraction of prosthesis. To ensure that reoperations are coded correctly the operating unit sends a copy of the medical records to the SHAR for manual centralized imputing.

The SPDR was introduced July 1, 2005 and is a register to which all pharmacies in Sweden are obliged to report and automatically include all outpatient prescribed drugs dispensed in Sweden. The SPDR includes information regarding the drug, the amount prescribed, the amount dispensed, the date of prescribing and dispensing, the instruction from the prescribing doctor, the level of care and the speciality of the prescribing doctor etc. The SPDR is based on the personal registration number and can therefore be connected to other national healthcare and quality registries in order to evaluate for example postoperative infections [10].

The study was given ethical approval by the Regional Ethical Committee in Gothenburg on 11 October 2010 (ref. 553–10).

All operations between July 1, 2005 and December 31, 2008 reported to the SHAR for a primary THR were included in the study (n = 49,219). All diagnoses, bilateral arthroplasties and all types of implants regardless of the method of fixation were included. By using the patient’s personal registration number, the cohort was then matched with the SPDR for all dispensed antibiotic prescriptions between July 1, 2005 and December 31, 2010.

**Figure 1:**
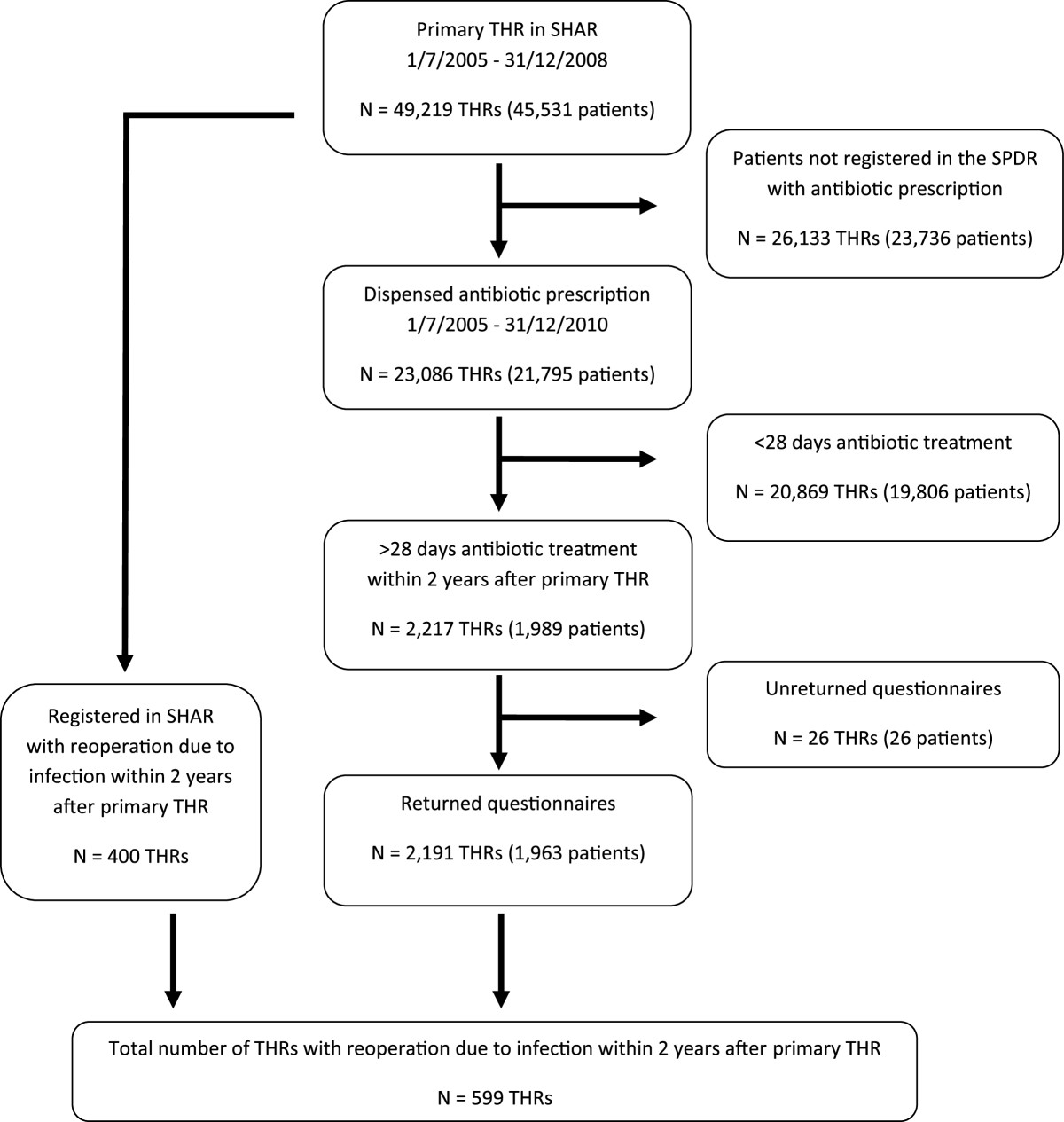
**Study flowchart.** (THR = Total Hip Replacement, SHAR = Swedish Hip Arthroplasty Register, SPDR = Swedish Prescribed Drug Register).

We limited the search by only including the dispensed amount of antibiotics suggesting a continuous outpatient medication for at least 4 weeks of selected antibiotics with Anatomic Therapeutic Chemical codes (ATC-codes) J01, J04 and P01 (Table 1). We limited the observation time to 2 years after the primary THR for each patient. Antibiotics where the instructions specifically indicated treatment for other infection (e.g. urinary tract infection, pneumonia) than a wound or prosthesis related infection were excluded.By matching the two registries we found that 1,989 patients with 2,217 THRs had been prescribed and dispensed >4 weeks of antibiotic treatment within the first 2 years after the primary THR. A questionnaire for each of the 2,217 THRs, including a list of dispensed antibiotics, was sent to a doctor at the primary operating unit (76 different units) to complete and return (Figure 1).

**Table 1:** **Number of tablets/doses required for 4 weeks of continuous treatment**

Antibiotic	ATC-code	Daily dose	4 weeks treatment
Amoxicillin	J01CA04	750 mg 1×3	84
Amoxicillin and clavulanic acid	J01CR02	875/125 mg 1×2	56
Azitromycin	J01FA10	250 mg 1×1	28
Cefadroxil	J01DB05	1 g 1×2	56
Cefalexin	J01DB01	3 g 1×2	56
Ceftriaxon	J01DD04	2 g 1×1	28
Ceftributen	J01DD14	400 mg 1×1	28
Cefuroxim	J01DC02	250 mg 1×2	56
Ciprofloxacin	J01MA02	250 mg 1×2	56
		500 mg 1×2	56
		750 mg 1×2	56
Daptomycin	J01XX09	400 mg 1×1	28
	J01DH03	1 g 1×2	28
Erytromycin	J01FA01	250 mg 2×2	112
Fenoximetylpenicillin	J01CE02	1 g 2×3	168
Flukloxacillin	J01CF05	500 mg 2×3	168
		750 mg 2×3	168
Fusidic acid	J01XC01	250 mg 2×3	168
Klaritromycin	J01FA09	250 mg 1×2	56
Klindamycin	J01FF01	300 mg 1×2	56
Levofloxacin	J01MA12	500 mg 1×1	28
Linezolid	J01XX08	600 mg 1×2	56
Lorakarbef	J01DC08	200 mg 1×2	56
Metronidazole	P01AB01	400 mg 1×3	84
Moxifloxacin	J01MA14	400 mg 1×1	28
Norfloxacin	J01MA06	400 mg 1×2	56
Rifampicin	J04AB02	600 mg 1×1	28
Roxitromycin	J01FA06	150 mg 1×2	56
Sulfametoxazol and trimetoprim	J01EE01	160 mg/800 mg 1×2	56
Teikoplanin	J01XA02	400 mg 1×1	28
Telitromycin	J01FA15	400 mg 2×1	56
Vancomycin	J01XA01	1 g 1×2	56

(ATC-code = Anatomic Therapeutic Chemical classification-code).

In the questionnaire the receiving doctor were asked to check each patient’s medical records and report if the THR had been reoperated due to infection within 2 years. If so, the type of procedure (wound debridement, irrigation of the prosthesis, exchange of one or more parts, or extraction of the entire prosthesis) was requested (hereafter called questionnaire data).

Of the 2,217 questionnaires sent out 2,191 (99%) were returned and all orthopaedic clinics contributed. 4 of the patients had incorrectly been registered in the SHAR as primary THR when in fact they had a revision THR. These cases were excluded reducing the final study sample to 2,187 THAs in 1,959 patients.

The SHAR reoperation database was searched for all reoperations due to infection within 2 years after the index THR of all primary THRs between July 1, 2005 and December 31, 2008 (hereafter called SHAR reoperation data).

Each THR was studied separately as one patient could have more than one THR. Each reoperated THR was only counted once even though it might have been reoperated several times.

### Statistics

The cumulative incidence of reoperation due to infection was calculated by dividing the number of reoperated THRs (in both questionnaire and SHAR reoperation data) by the total number of primary THRs in Sweden between July 1, 2005 and December 31, 2008 excluding the THRs in patients that died without reoperation within 2 years after the primary THR (n = 47,358). Sensitivity, specificity, negative predictive values (NPV), positive predictive values (PPV) and measurement agreement according to Cohen’s Kappa [11] was calculated for the SHAR reoperation data compared with the questionnaire data. We used the IBM SPSS Statistical software version 21 for Windows.

## Results

**Figure 2:**
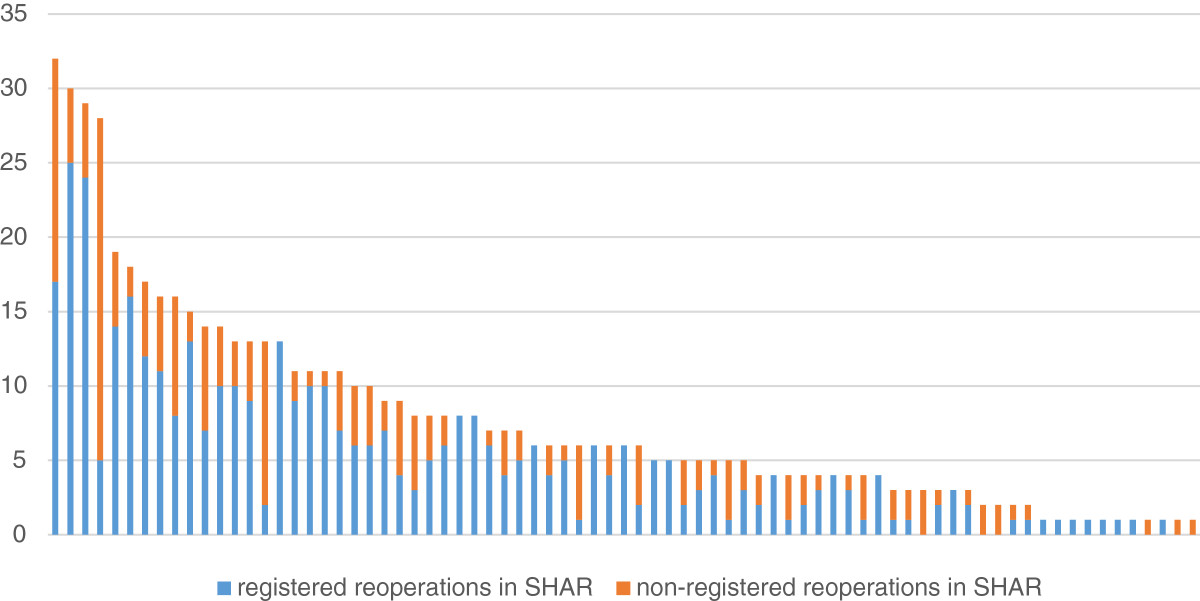
**Number of registered and non-registered reoperations for each operating unit.** Each bar, representing one operating unit, is divided into registered and non-registered reoperations. All reoperations are due to infection and within 2 years after primary Total Hip Replacement.

A total of 599 primary THRs were identified by the questionnaire data and the SHAR reoperation data making the cumulative incidence of reoperation due to infection 1.3%. 302 (50%) of the reoperated THRs were found in both the questionnaire and SHAR reoperation data and the measurement agreement according to Cohen’s Kappa was 0.67 (Table 2). The THRs not recorded as reoperated due to infection in the SHAR (n = 199, 67%) included all types of reoperations (Table 3). The number of non-registered reoperations for the individual primary operating units ranged between 0 to 100% and was not associated to the total number of reoperated infections by each unit (Figure 2).

**Table 2:** **The number of THRs with a reoperation due to infection found by either of the two methods including calculated sensitivity, specificity, positive predictive value (PPV), negative predictive values (NPV) and method agreement by Cohen’s Kappa**

		Questionnaire data		
		Yes	No	
**SHPR reoperation data**	Yes	**302**	**98**	400
	No	**199**	**46759**	46958
		501	46857	47358
	Sensitivity		0.60	
	Specificity		0.99	
	PPV		0.76	
	NPV		0.99	
	Agreement		0.67

**Table 3:** **Type of reoperation due to infection within 2 years after primary THR, total numbers and non-registered cases in the Swedish Hip Arthroplasty Register (SHAR)**

Type of reoperation	Total		Non-registered in SHAR	
	n	%	n	%
**Reoperation without revision of prosthesis**				
*Wound revision*	*103*	*17.2*	*58*	*29.1*
*Irrigation of prosthesis*	*212*	*35.4*	*79*	*39.7*
**Total**	**315**	**52.6**	**137**	**68.8**
**Revision of prosthesis**				
*Exchange of parts or entire prosthesis*	*173*	*28.9*	*45*	*22.6*
*Extraction (with or without spacer)*	*111*	*18.5*	*17*	*8.5*
**Total**	**284**	**47.4**	**62**	**31.2**
**Total**	**599**	100	**199**	**100**

## Discussion

This study presents an external validation of the SHAR reoperation database in terms of reoperation due to infection and shows that 67% of the reoperated infections are reported to the register.

The strengths of this study are the national coverage of the SPDR and the number of medical records studied and returned questionnaires by the operating units. The study of medical records is also likely to give a more reliable validation compared to other methods such as procedure codes in administrative databases (that lack specificity of side) or questionnaires filled in by patients.

Limitations of the study design are that the validation by the SPDR requires that the patient had been prescribed and dispensed outpatient antibiotic treatment for the infection. Therefore patients that died before discharge or were never prescribed or complying to antibiotic treatment were not identified in the SPDR. Information on patients that had no follow-up at the primary operating unit could also be missing in the questionnaire as well as the risk of reporting bias by the doctors doing the medical records review. It is therefore possible that the incidence in this study is an underestimation of the true reoperation incidence.

The low completeness of registered reoperations due to infection in the SHAR varied greatly between the operating units, but seems to be a general problem of the Swedish orthopaedic community. The reason for the low completeness is probably multifactorial but one important reason might be that reoperations due to infection with no or minor implant exchange are performed in an acute setting and could therefore deviate from the standard routine reporting. Another reason might be that other orthopaedic quality registries (e.g. the Swedish Knee Arthroplasty Register) only record revisions (implant exchange or extraction) and not all reoperations introducing an uncertainty in the reporting routines to the SHAR [12]. The large number (68.8%) of non-recorded reoperations without implant exchange or extraction supports this (Table 3). There is also a possibility that units taking care of their own complications deliberately fail to report reoperations to the SHAR in order to improve their results in the SHAR annual report. However, the high compliance to our questionnaire, where all operating units participated speaks against this presumption. There are advantages of a central monitoring system like a national arthroplasty register as the follow up is not limited to the primary operating unit. This is likely the most important reason to why 98 of the reoperations identified via the SHAR reoperation data were missing in the questionnaire data (Table 2).

Arthroplasty register data has been used to study the change of infection rates after total hip arthroplasty [5, 6]. National arthroplasty registers however only capture infections that are reoperated (and in some registries only the revised ones) and not the infections treated by antibiotics only [13–15]. In Sweden 9% of deep early and delayed periprosthetic infections are treated with antibiotics without surgical intervention [16]. The change in infection rates can therefor reflect a change in reoperation policy or the change in arthroplasty modularity making it easier to revise the prosthesis by exchange of head or acetabular liner. The low completeness found in this study also imply that change in reporting routines could have a large impact on the change in infection rates in register data. That only 47.4% of the reoperations due to infection in this study were revisions, implies that especially those arthroplasty registers that only record revisions seem less reliable when studying infection rates as revisions are dependent on the modularity of the prosthesis (Table 3). In contrast to previous arthroplasty validation studies that have found extraction of prosthesis (i.e. Girdlestone procedure) due to infection to be the most common error in registration, we found this procedure to be the most reliable of the four categories used by us [1, 3]. The conclusion is that the SHAR data cannot with certainty be used as an estimation of the total infection burden after THR.

Although the data in this study only investigates the number of surgically treated THRs due to infection (i.e. excluding the non-surgically treated), knowledge about the true proportion of reoperations is important for both clinicians and patients when estimating risks before planning a primary THR.

The validity of a total joint replacement register is dependent on the coverage and completeness of the register. The coverage (i.e. the number of operating units that report to a register) is important, but the figures can be misleading if the completeness (i.e. the number of correct registrations on an individual level) is low at some or all operating units. This can lead to both an underestimation of the true total incidence of reoperations and to an incorrect relative incidence between the individual reporting units. It is therefore important to evaluate both coverage and completeness so that the results can be interpreted with justice.

The control of completeness of primary THRs is standardized in the SHAR by an annual matching of the registered primary THRs in the register with the Swedish National Patient Register (NPR) procedure codes via the patients’ personal registration number. To guarantee high quality of the reoperation data in the database the operating unit sends a copy of the medical records to the SHAR for manual centralized imputing. Subsequent interventions, however, are more difficult to automatically validate by the same method as primaries as there are many different possible procedure-codes and as the NPR lacks laterality (left or right hip), making numerous combinations possible. To control that all subsequent procedures are reported, the SHAR has newly started a monitoring system by site visits to the operating units where the SHAR data is compared to local administrative data and medical records.

## Conclusions

The completeness of the reoperation data in the SHAR questions whether data from arthroplasty registers that record reoperations or revisions as complication outcome can be used in order to evaluate both non-surgically and surgically treated infection rates after THR. An open publication along with feedback to the reporting units will hopefully improve reporting routines in the future.

## Authors’ original submitted files for images

Below are the links to the authors’ original submitted files for images. Authors’ original file for figure 1 Authors’ original file for figure 2

## Abbreviations

THR: Total Hip Replacement
SHAR: Swedish Hip Arthroplasty Register
SPDR: Swedish Prescribed Drug Register
NPR: Swedish National Patient Register.
