# Supplementary figures and images for: Validation of reoperations due to infection in the Swedish Hip Arthroplasty Register
Source: BMC Musculoskelet Disord. 2014 Nov 19;15:384. doi: 10.1186/1471-2474-15-384 (PMC4247680; doi:10.1186/1471-2474-15-384)

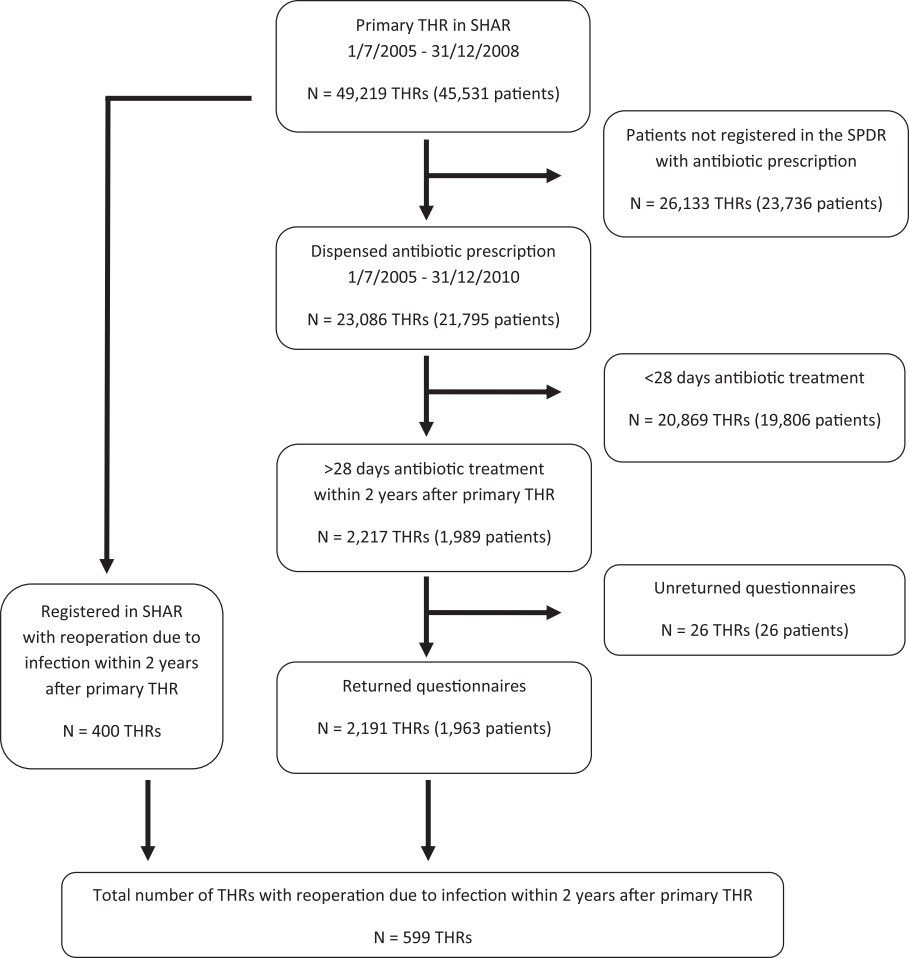

Supplement: Supplementary file 1 — Authors’ original file for figure 1 [file 12891_2014_2332_MOESM1_ESM.pdf]

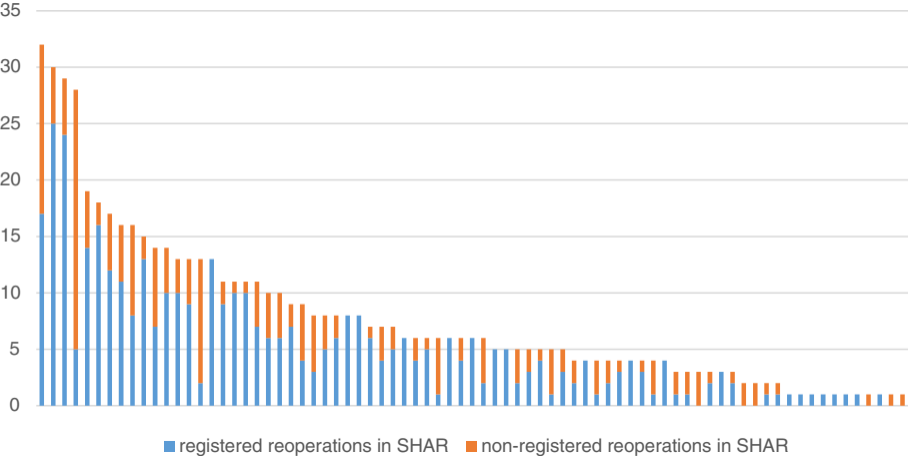

Supplement: Supplementary file 2 — Authors’ original file for figure 2 [file 12891_2014_2332_MOESM2_ESM.pdf]
